# Comparing Different Approaches for Mapping Urban Vegetation Cover from Landsat ETM+ Data: A Case Study on Brussels

**DOI:** 10.3390/s8063880

**Published:** 2008-06-10

**Authors:** Tim Van de Voorde, Jeroen Vlaeminck, Frank Canters

**Affiliations:** Vrije Universiteit Brussel, Department of Geography, Cartography and GIS Research Unit, Pleinlaan 2, B-1050 Brussels, Belgium; E-Mails: tvdvoord@vub.ac.be (T.V); vlaeminck.jeroen@gmail.com (J.V.); fcanters@vub.ac.be (F.C.)

**Keywords:** urban vegetation cover, spectral mixture analysis, multi-layer perceptrons

## Abstract

Urban growth and its related environmental problems call for sustainable urban management policies to safeguard the quality of urban environments. Vegetation plays an important part in this as it provides ecological, social, health and economic benefits to a city's inhabitants. Remotely sensed data are of great value to monitor urban green and despite the clear advantages of contemporary high resolution images, the benefits of medium resolution data should not be discarded. The objective of this research was to estimate fractional vegetation cover from a Landsat ETM+ image with sub-pixel classification, and to compare accuracies obtained with multiple stepwise regression analysis, linear spectral unmixing and multi-layer perceptrons (MLP) at the level of meaningful urban spatial entities. Despite the small, but nevertheless statistically significant differences at pixel level between the alternative approaches, the spatial pattern of vegetation cover and estimation errors is clearly distinctive at neighbourhood level. At this spatially aggregated level, a simple regression model appears to attain sufficient accuracy. For mapping at a spatially more detailed level, the MLP seems to be the most appropriate choice. Brightness normalisation only appeared to affect the linear models, especially the linear spectral unmixing.

## 1. Introduction

Urbanisation has reached an important milestone in 2008: more than half of the earth's population now lives in urban areas [1]. The estimated annual urban population growth rate of 1.78% is nearly twice as fast as that of the global population. If this trend continues, 5 billion people out of a total world population of 8.1 billion will reside in cities by 2030. While urban growth is strongest in developing regions of Africa and Asia, with annual urban population growth rates of 4.58% in Sub-Saharan Africa and 3.82% in South-East Asia, most industrialised countries in Europe and the Americas already have urban populations of 80% or more [2]. It is no surprise then, that cities are conflict zones between economic growth, society and the environment. They suffer from many environmental problems: air pollution, traffic jams, high levels of ambient noise, empty houses and derelict lands, which undermine the quality of life of city dwellers and imprint a negative view of urban life. This in turn drives urban residents to move closer towards the edges causing urban sprawl with increased traffic, decay of city centres and other self reinforcing problems as a consequence [3]. Ensuring high quality urban environments is therefore an important aspect of the EU's strategy for sustainable development [4] and has led to an increased interest in monitoring urban environments in terms of quality of life and urban growth. Urban green is an important contributor to the quality of urban environments. Vegetation within urban areas has been valued since long given that parks, gardens and avenues of trees have been traditional features of town planning. From an ecological point of view urban green has a positive effect on biodiversity and air quality, mitigates the urban heat island effect, and allows precipitation to seep into the ground, reducing flood risk. Green areas also provide recreation for citizens, improve the aesthetic appeal of neighbourhoods and increase property values. As a consequence, information on the abundance and spatial distribution of urban vegetation is of great value to support the development of sustainable urban policies, and may form a key component of urban quality of life indicators. Many urban agglomerations already have some form of urban green database at their disposal, often even containing individual trees. Such databases are mostly either incomplete in a sense that they do not cover public as well as private green areas, they are not frequently updated or they are incompatible in one way or another with other cities' databases. The Brussels Institute of the Environment, for instance, composed a database of green areas at the end of the nineties but did not update it since with respect to green areas that are not under their management, such as private gardens or public green areas maintained by the communities [5].

Remotely sensed data from earth observation satellites may provide a significant contribution to urban green monitoring. Procedures to extract such information from digital imagery are generic and lead to results that are comparable among different urban areas, which is ideal for monitoring purposes at regional, national or supranational scales. Satellite images also make it easier to conduct frequent updates, and even allow extracting historic information on urban vegetation to study vegetation trends. Despite the currently available high resolution satellite or airborne images, which allow a detailed mapping of urban green at local scales, the advantages of medium resolution data should not be overlooked. First of all, the Landsat program has been collecting images since 1972 and its extensive archive provides a unique historic perspective on urban growth and changes in urban vegetation. Medium resolution images also have a larger footprint, are cheaper and often even available for free, which makes them ideally suited for synoptic mapping. Furthermore, a detailed mapping of individual vegetation elements, which would call for high resolution data, is not required for many applications related to sustainable urban management at strategic or higher levels of planning. Despite all these advantages, however, the relatively low spatial resolution of a medium resolution satellite sensor may lead to low mapping accuracies because the instantaneous field of view (IFOV) often contains different types of land cover, especially in urban areas [6]. The traditional supervised image classifiers used to derive land-cover maps from digital images assign pixels individually to a single pre-defined target land-cover class, and will run into difficulties when dealing with such mixed pixels. Spectral mixture analysis addresses this problem by unmixing (deconvolving) each pixel spectrum into fractional abundances of its surface constituents or endmember spectra [7]. Many different approaches exist to model a composite spectrum as a mixture of pure land-cover types, but they all can be roughly divided into linear and non-linear models [8, 9]. Linear spectral mixture analysis (LSMA) is the most common approach. It is based on a physical model that assumes that the spectral signature of a pixel is a linear combination of end-member spectra. Linear regression is a well known statistical technique that may also be used to estimate fractional cover of a single land-cover type [10, 11]. In its most simple form, only one independent variable is used. More complex models employ multiple independent variables. Next to linear approaches, many different types of non-linear unmixing models have been proposed. Some are based on multi-layer perceptrons [12], or other types of neural networks such as ARTMAP and ART-MMAP [13, 14] or self-organizing maps [15]. Others draw on regression trees [16, 17] or fuzzy classifiers [18]. Of all these non-linear unmixing approaches, models based on multi-layer perceptrons (MLP) are the most widely used [9]. Spectral unmixing techniques have been frequently and successfully applied for vegetation mapping at medium resolution. Several authors applied regression analysis between NDVI and known vegetation fractions to estimate fractional vegetation cover [19]. This approach is reliable and efficient [20], but other authors found that vegetation estimates derived from spectral mixture modelling appear less sensitive to background soil reflectance [21, 22]. Other research indicated that MLP performs better than unconstrained least squares LSMA, for instance in an experiment to estimate oak wood crown closure from Landsat TM data [23].

The objective of this research was to efficiently extract information on urban vegetation abundance from medium resolution data so it may serve as basic data for urban environmental monitoring in the Brussels Capital Region. For this purpose, we compared three unmixing approaches: linear regression analysis (LR), linear spectral unmixing (LSMA) and multi-layer perceptrons (MLP). Next to a quantitative validation on a per-pixel basis, fraction estimates were aggregated to meaningful urban spatial units, i.e. neighbourhoods to make the comparison between the models more relevant in an urban planning context.

## 2. Study area and data

### 2.1. Study area

**Figure 1.**
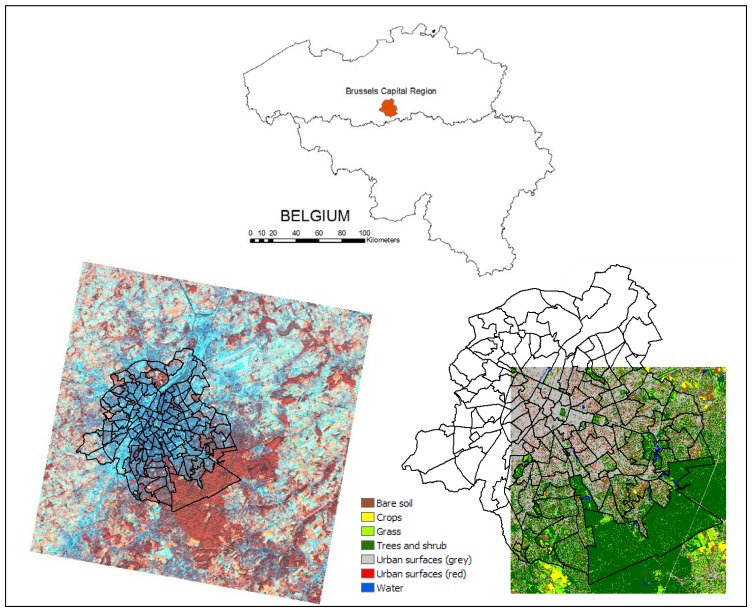
The Brussels Capital Region, Belgium (top). Landsat ETM+ image (left) and high resolution land-cover classification (right) projected on a map showing the city's neighbourhoods.

The focus of our study was the Brussels Capital Region, centrally located in Belgium (figure 1) with a size of 161.78 km^2^ and a registered population of 1 031 215 on January 1^st^ 2007 [24]. Urbanisation in the capital region continues at a steady pace after a period of rapid growth in the eighties. Between 1980 and 2003, the amount of built-up land increased by 13% while the un-built areas decreased by 17%. [25].

Brussels is a green city compared to some other European capitals. According to an inventory made in 1999 by the Brussels Institute of Environmental Management (BIM) the city has 8563 hectares of green areas, which is 53% of the total area. This includes public parks and recreation areas, unused land, forests, cemeteries, private gardens and estates, with private green representing about 42% of all urban green. These areas are, however, unequally distributed. The neighbourhoods near the edge of the capital region are best served with an urban green cover of 30% to 70%, while the more central areas consist of only 10% or less vegetation [26].

### 2.2. Image and ancillary data

The image data we used in this study is a subset (1164 lines by 1164 samples) of a Landsat ETM+ (level 1G) image acquired on October 18^th^ 1999 (path 198, row 25). The subset contains the entire city and a good part of its surroundings (figure 1). The digital numbers (*DN*) of the ETM+ image were converted to apparent reflectance according to the formulas and calibration parameters presented by The Landsat 7 Users Handbook [27]. Homogeneous atmospheric conditions in the image were assumed, so no atmospheric corrections were performed.

Reference data for training and validating the sub-pixel classification models was obtained from an existing high resolution land-cover classification, which was derived from a multispectral (4m resolution) and panchromatic (1m resolution) Ikonos image acquired on June 8^th^ 2000. The land-cover map was created by a MLP classification in combination with a rule-based post-classification and shadow-removal approach, and has a reported accuracy (kappa index) of 0.95 [28]. It covers a large part of the city, from the centre towards the south-east (figure 1).

The ETM+ image was geometrically co-registered to the land-cover data by a first-order polynomial transformation. The RMS error on an independent set of control points was 5.78 m, which implies that on the average the geometric shift between land-cover map and ETM+ data is less than 4% of an ETM+ pixel's area. This is considered an adequate result. The bias this will cause on proportion estimates will certainly be much less than errors inherent to the sub-pixel classification model.

### 2.3. Training and validation data

Training and validation samples to build and test the three sub-pixel models were derived from aggregating the classes of the reference land-cover classification to three target classes: impervious surfaces, vegetation and bare soil. Water was not included as a separate class because few water bodies are present in the study area. The location of those that are present (a canal and some ponds) is well known and they were masked out from the study area. Parallel to this thematic aggregation, a spatial aggregation of the 1m high-resolution classification to 30m grid cells was carried out to obtain land-cover proportions at ETM+ resolution. In total, 3037 randomly chosen ETM+ pixels were selected for training, and 5919 different pixels were independently chosen for validation. This set represents the continuum of class mixtures for each of the three classes. To ensure that no sample pixels were included for which the land cover had changed in the time span between the acquisition of the Landsat image and the Ikonos data from which the land-cover map was derived, a temporal mask was used. This mask indicates change pixels and was created by identifying outliers in the assumed linear relationship between the normalized difference vegetation index (NDVI) of the ETM+ pixels and the average NDVI of the overlapping Ikonos pixels [29].

## 3. Methods for estimating per-pixel vegetation fractions

### 3.1. Linear spectral mixture analysis

Linear spectral mixture analysis (LSMA) is a common approach to sub-pixel classification whereby a pixel's observed radiance is modelled as a linear combination of spectrally pure “endmember” radiances. Each endmember contributes proportionally to the overall spectral response according to its relative abundance within the sensor's instantaneous field of view (IFOV) [30-32]. To estimate the fractional cover of each endmember within a given pixel, the following equation has to be solved for all image bands simultaneously, using a least squares approach:

(1) $$R_{b}=\sum_{i=1}^{n}f_{i}r_{i,b}+e_{b}$$

where *R_b_* is the reflectance of the pixel for band *b*, *f_i_* is the proportion of endmember *i* within the pixel, *r_i,b_* is the reflectance of endmember *i* for band *b*, *n* is the number of endmembers and *e_b_* the error of fit for band *b* [33]. Inverting this system of mixing equations to retrieve endmember fractions that best fit the observed mixed reflectances implies determining the optimal location of endmembers in feature space.

While LSMA was first applied in the field of mineralogy, it soon found its way to land surface and vegetation mapping where it has been used to derive fraction images representing land-cover proportions within each pixel [34-36]. More recently, LSMA has received quite some attention in studies that aim to characterise urban environments [37-42]. For this purpose, the VIS model is a useful conceptualisation of the urban environment because it allows representing any urban area by three physical components: vegetation (V), impervious surfaces (I) and soil (S), in addition to water [43]. If these components could be unambiguously represented as endmembers in feature space, fractions derived from unmixing an urban area would allow to position urban pixels in the VIS triangle. This in turn would make it possible to analyze urban morphology, form and function starting from medium resolution satellite imagery. However, not all pure vegetation, impervious surfaces or bare soil classes occupy extreme positions in feature space and can, as such, not be directly used as endmembers for unmixing.

**Figure 2.**
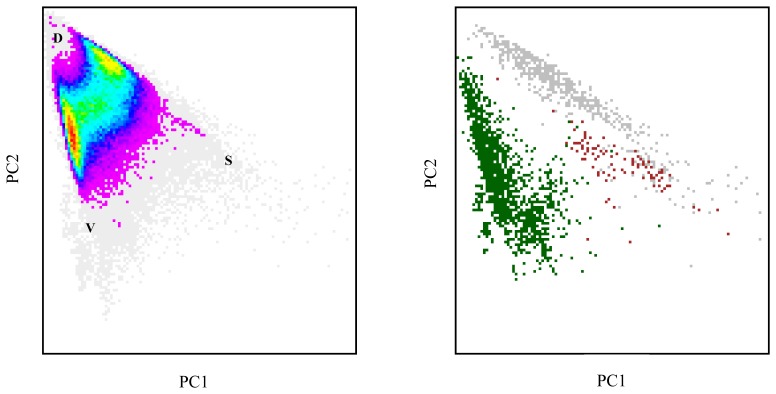
Landsat ETM+ feature space made up by the first and second principal components (left) and position of pure pixels within that feature space (right). Colours on the left graph indicate pixel frequencies ranging from very low densities (grey) to high densities (red and yellow). Colours on the right graph indicate pure pixels vegetation (green), impervious (grey) and bare soil (brown).

The Landsat ETM+ mixing space of Brussels has a similar appearance to what has been reported for other urban areas [44]. We can examine it more closely by performing a principal component analysis on the image data and visualising the location of all image pixels in a two-dimensional graph defined by the first and second principal components. This produces a typical triangular-shaped distribution, with the apexes of the triangle corresponding to true biophysical endmembers representing high albedo substrate (S), bright vegetation (V) and dark surfaces (D) [45] (figure 2, left). Any pixel falling inside the convex hull circumscribing the apexes can be considered as a mixture of these three components [46]. Determining the position of pure vegetation, impervious surface and bare soil pixels in this graph can be achieved by using the reference dataset derived from the high-resolution land-cover map, for which all ETM+ pixels that consist of over 95% of either vegetation, impervious surfaces or bare soil are considered pure (figure 2, right). Although pure soil pixels are somewhat clustered together in the mixing space, they clearly coincide with pure impervious pixels near the substrate endmember. In contrast with studies carried out on other areas [8, 37], bare soil was also not present as a separate endmember if the mixing space was visualised with the third principal component. Man-made impervious surfaces and exposed soils may indeed be spectrally very similar, depending on the soil type and characteristics on broadband image data [8]. For instance, Van de Voorde *et al.* reported high levels of spectral confusion between exposed soils near Brussels and red-clay roof tiles, very common in the city [28]. This will complicate the unmixing process and will lead to some degree of confusion between these two land-cover types if they are chosen to represent endmembers of a VIS unmixing model. Furthermore, endmembers in urban areas are spectrally variable because of brightness differences [47]. This is clearly indicated by the position of pure pixels all around the edges of the mixing space. Pure vegetation pixels are mostly located on the vegetation – dark axis, indicating binary mixing between these two endmembers. Darker vegetation types such as trees are located closer to the dark endmember, while brighter vegetation types such as grass or crops are typically found closer to the vegetation endmember. Binary mixing on the “grey axis” between the dark and substrate endmembers represents different types of urban surfaces, e.g. asphalt versus concrete, while binary mixing on the vegetation – high albedo substrate axis is extremely rare [45]. This further complicates the direct use of the VIS ternary as an appropriate model for unmixing.

Wu (2004) pointed out that while individual spectra for pure vegetation-impervious-soil pixels show significant brightness variation, their spectral shape is similar [47]. He therefore proposed the normalisation method shown in equation (2) to highlight shape information while minimizing the effect of absolute brightness differences.

(2) $${\bar{R}}_{b}=\frac{R_{b}}{\mu }\times 100$$

with

(3) $$\mu =\frac{1}{N}\sum_{b=1}^{N}R_{b}$$

where *R̄_b_* is the normalized reflectance for band *b* in a pixel, *R_b_* is the original reflectance for band *b* and *N* is the number of bands (6 for Landsat ETM+).

**Figure 3.**
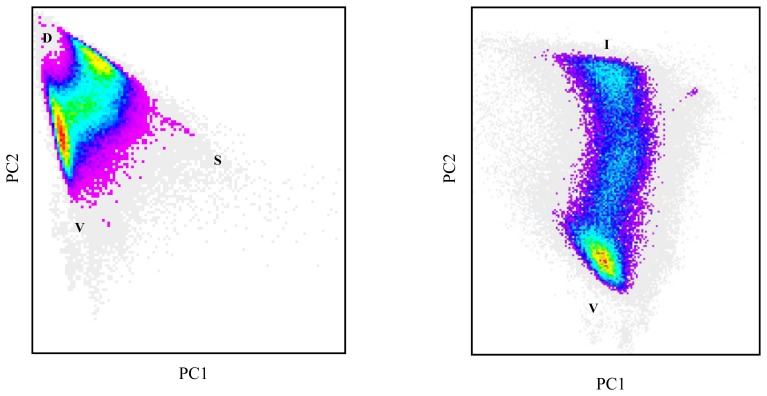
Landsat ETM+ feature space made up by the first and second principal components before (left) and after brightness normalisation (right). Colours indicate pixel frequencies ranging from very low densities (grey and magenta) to high densities (red and yellow).

Because the aim of this study is to isolate a general vegetation endmember rather than distinguishing different types of vegetation, brightness normalisation may help to reduce spectral variability and improve vegetation proportion estimates. This is clearly demonstrated by the impact normalisation has on the shape of the mixture space (figure 3), which becomes more elongated with pure vegetation pixels on one side and impervious surfaces on the opposite side. A model with two endmembers (vegetation versus non-vegetation) therefore appears to be best suited to estimate vegetation fractions.

The quality of the obtained fraction images depends greatly on the selection of suitable endmembers [48]. While many different approaches have been suggested, endmembers extracted directly from the image are most often used because they can be easily obtained and directly related to surface components in the scene [49, 50]. A frequently used approach to obtain endmembers is by selecting extreme pixels in a feature space visualisation by means of high order principal components [46]. As a first approach to linear unmixing in this study, three endmember (V, I, S) and two endmember (V, I+S) models were developed with and without brightness normalisation. Endmember locations for unmixing were determined from the training data by averaging the position of the pure pixels representing each endmember in feature space. As an alternative for the VIS model, the high albedo substrate, vegetation and dark surfaces (SVD) biophysical model can be directly used for determining the proportion of illuminated vegetation [51]. In this study, we manually selected the three endmembers of the SVD model on the graph formed by principal components 1 and 2. For vegetation and dark surfaces this was relatively easy given the fact that the mixing space tapers near those two edges. The divergent behaviour of the mixing space near substrate makes the selection of that endmember less straightforward. However, with respect to estimating vegetation proportions, the choice of the high albedo substrate endmember in a SVD framework is probably less crucial than positioning the other two. Vegetation fractions are less sensitive to moderate variability of larger fractions of high albedo substrate because binary mixing between vegetation and substrate is rare [45]. A major drawback of the SVD model is that the estimated vegetation fractions cannot directly be linked to actual vegetation fractions on the ground, because high fractions of the vegetation endmember reflect bright vegetation. Darker vegetation types such as trees are represented as mixtures of the dark and vegetation endmembers. Directly relating the vegetation fraction of a SVD unmixing to percentage vegetation cover therefore results in underestimations of darker vegetated areas. To account for these underestimations in the present study, the output of the vegetation fraction of the SVD model was modified. For each pixel, the actual vegetation cover was calculated by adding the vegetation fraction to the dark endmember fraction if the estimated vegetation fraction was higher than a threshold value, otherwise the original value was retained. This threshold was required to remove water and other dark, non-vegetation pixels from the low-albedo fraction image. A comparable approach was used by Lu and Weng [52] to separate impervious surfaces from bright soil in the high albedo fraction image. In this study, the threshold was set to 0.20 by minimising the error of estimated vegetation cover on the training dataset.

### 3.2. Linear regression analysis

Regression analysis is a common statistical technique to examine the relation of a dependant or response variable to specified independent or explanatory variables without relying on any assumptions about underlying processes [53]. In linear regression analysis, the relation between the dependant variable *Y* and the explanatory variables *X_i_* is assumed to be a linear function:

(4) $$Y=\beta_{0}+\sum_{i=1}^{n}\beta_{i}X_{i}+e$$

where *β*_0_ is the intercept, *β_i_* the regression coefficient of the *i^th^* independent variable, *n* the total number of independent variables, and *e* a random error term that represents the unexplained variation in the dependant variable. The coefficients are usually estimated with the least squares approach, in which the error term *e* is minimised.

The linear regression model can be applied to estimate vegetation abundance within a pixel by assuming a linear relationship between a pixel's vegetation fraction (response variable) and the spectral bands of Landsat ETM+ (explanatory variables). A choice has to be made about which bands should be included as variables in the regression model because they each do not explain the observed variance of the dependant variable to the same extent. Some of them may even be redundant to predict vegetation fractions from spectral values. To examine which bands should be included in the model, a stepwise regression approach can be used in which at each step a variable is added or removed according to its explanatory value to the model. The observations of vegetation fractions that are required to develop the regression model were derived from the available high-resolution land-cover classification by counting the number of high-resolution vegetation pixels inside each of the 3037 randomly selected reference ETM+ pixels. The resulting regression model was applied on the entire Landsat image. Like for the other unmixing approaches, the accuracy of the predicted vegetation fractions was estimated on the independent validation set consisting of 5919 ETM+ pixels. Because the regression model is not constrained, predicted fractions may be negative or larger than 1. To account for this, estimated fractions that fell outside the theoretically possible range were changed to either 0 or 1.

### 3.3. Unmixing with neural networks

A multi-layer perceptron (MLP) using the back-propagation learning rule [54] was used as a third approach to predict vegetation abundance from observed spectral radiances. As opposed to linear regression or linear mixture analysis, the MLP makes no assumptions about the nature of the relationship between sub-pixel proportions and spectral response. It rather adapts itself to the training samples and forms decision boundaries in feature space that can be applied on unknown data. Depending on the size of the MLP (i.e. number of hidden nodes), more complex boundaries may be formed than the hyperplanes defined by the linear equations of the linear mixture analysis approach. Because the mixing problem is often non-linear [12], using a MLP for unmixing may lead to higher accuracies depending on the nature of the mixture problem. This was demonstrated by several authors. For instance, Atkinson *et al.* [55] applied a MLP model to unmix AVHRR imagery and noted a superior performance compared to the linear unmixing model and a fuzzy c-means classifier. Van de Voorde et al. [29] compared linear spectral unmixing to a MLP for impervious surface mapping and found that the MLP was more accurate, and that this could be explained by a better representation of the mixture space by the MLP. Another advantage of using MLP for unmixing is that no pure endmembers, which are often difficult to obtain, need to be defined. Opposed to these potential advantages lies the drawback that the user has to make several design choices, which can have a significant effect on MLP performance: network architecture (number of hidden layers/neurons), parameterisation of the back-propagation algorithm, input/output coding, weight initialisation, etc. [56]. Taking the complex and often ambiguous parameterisation into consideration, we used NeuralWare's Neuralworks Predict® to develop the MLP for sub-pixel classification of vegetation. This commercial software package offers the user a set of semantic design choices which optimally set or determine the parameters required to train a neural network, such as the number of hidden nodes, learning rate, number of epochs, etc. [57]. In terms of input coding, the software transforms the variables according to a range of available functions, and selects the most appropriate ones with a search technique based on genetic algorithms. The architecture is not fully fixed a priori, but Predict draws instead on a constructive method to determine the number of hidden nodes, referred to as cascade learning [58]. With this approach, hidden units are added one or a few at a time in such way that each new node receives input from both the input layer as well as from the previously established hidden nodes. The construction process is stopped when performance on an independent test set shows no improvement.

Using NeuralWorks Predict®, we developed three-, two- and one-endmember MLP models to estimate sub-pixel vegetation fraction, using the full set of 3037 randomly selected reference ETM+ pixels for network training. Also in this case results obtained with models developed on original reflectance data were compared to results obtained with brightness normalised data.

## 4. Model validation

The three sub-pixel classification methods and their variations based on brightness normalisation, number and type of endmembers or independent variables used for model development produce 16 alternative predictions of vegetation abundance within the ETM+ pixels. The accuracy of these predictions can be assessed with the mean absolute error (*MAE_Veg_*) calculated from the set of 5919 Landsat ETM+ validation pixels (table 1). It quantifies the amount of error and can be interpreted as a mean error percentage. In addition, the mean error (ME*_Veg_*) was used to indicate a possible bias in the estimation of the vegetation proportions (over or underestimation).

(5) $${\text{MAE}}_{\text{veg}}=\frac{\sum_{j=1}^{N}\left|P\prime_{j}-P_{j}\right|}{N}$$

(6) $${\text{ME}}_{\text{veg}}=\frac{\sum_{j=1}^{N}\left(P\prime_{j}-P_{j}\right)}{N}$$

with

*N:* the total number of pixels in the validation sample
*P_j_*: the proportion of vegetation inside validation pixel *j*, derived from the high-resolution classification (ground truth)
*P′_j_*: the proportion of vegetation inside validation pixel *j*, estimated by the sub-pixel classifier

As expected, the LSMA model with the SVD endmembers has a very low accuracy (*MAE_Veg_* of almost 35%) because it underestimates dark vegetation types. The proposed modification to the SVD model's output overcomes this problem, as the mean absolute error of the vegetation cover estimate decreased strongly to 0.1270 (about 13%), and the error bias improved from -0.3246 (i.e. a severe underestimation) to near zero. This is a slightly better result than unmixing with VIS endmembers (0.1322). When the VIS model was implemented with the normalised data, the accuracy improved with about two percentage points to 0.1090. This demonstrates the impact of brightness differences on the performance of LSMA models. An unmixing model with only two endmembers (vegetation versus no vegetation) results in only slight improvements for normalised as well as not normalised data compared to the use of a three-endmember model (table 1). All unmixing models with the exception of the modified SVD model tend to slightly underestimate vegetation cover, as indicated by the negative error biases.

Results of the stepwise linear regression approach (table 2) show that the average error magnitude is 0.120 when all not normalised variables are included in the model. In that case the coefficient of determination, adjusted for the number of variables, indicates that about 81% of the observed variability in the vegetation fractions is accounted for by the model. If only the red and infrared bands are included, still 79% of the variability is explained, and an average error magnitude of 0.127 is attained. This is not unexpected, because the combination of the red band (low reflectance for vegetation) and the infrared band (high reflectance for vegetation) allows distinguishing vegetated from not vegetated surfaces, and is also used to calculate well-known vegetation indices like NDVI [59]. Other work already demonstrated a strong linear relationship between NDVI and sub-pixel vegetation fractions in urban scenes, albeit with certain restrictions in areas where the vegetation is mixed with dark non-vegetated types of land cover [60]. When only the infrared band is used as explanatory variable in the model, the error magnitude increases substantially to 0.208. A regression model based on the red and infrared band therefore seems to be a good choice, given its simplicity. When the stepwise regression is applied with the bands after brightness normalisation, the error magnitude drops by about 0.015. A major difference compared to the scenario without normalisation, however, is the high accuracy obtained with only the infrared band. This can be explained by the fact that brightness normalisation enhances the contrast between the near infrared band and the other bands. Without normalisation, the red band has to be included in order to obtain this contrast. The most accurate regression model uses the normalised red and green bands and the two middle infrared bands as independent variables. This model accounts for about 85% of observed variability. For the regression models there is also a general trend towards underestimation, but less pronounced than the trend observed for the VIS unmixing (table 1).

The *MAE_Veg_* values obtained with MLP models are lower than those of comparable scenarios for the other two unmixing approaches (table 1). The errors among the various MLP scenarios are also more similar. Brightness normalisation of the input data does not seem to have as much impact on the results as it does for the regression and linear unmixing models. Reducing the number of target classes, i.e. output nodes, to two (vegetation / no vegetation) or one (vegetation) also produces only marginally better results. The best MLP scenario is a model with a single endmember implemented on normalised data. In contrast to the other models, the ME*_Veg_* indicates that the MLP models tend to slightly overestimate vegetation fractions (i.e. positive error bias).

The unmodified SVD linear unmixing put aside, the largest error disparity across the 16 unmixing scenarios occurs between the normalised MLP with one endmember (0.095) and the not normalised VIS unmixing (0.132). The smallest error differences occur among the various MLP scenarios, which appear to yield almost identical results. Given these small absolute error ranges, a statistical test to assess the significance of the observed error disparities is indispensable. Because normality tests indicated that the error differences between the various scenarios were not normally distributed, the non parametric Wilcoxon signed-rank test was used [61, 62]. The most accurate scenario is the MLP with a single output node using spectrally normalised data. The Wilcoxon p value indicates, however, that the same model using not normalised data does not produce significantly different results. Among other MLP scenarios, the effect of normalisation is unsure as well. For the MLP models with two and three outputs, the effect of brightness normalisation is statistically significant. However, in the two-endmember case results obtained with normalisation are worse than without normalisation. All this suggests that brightness normalisation has little or no influence on the accuracy of a MLP based sub-pixel classification model. It does have a significant impact, however, on linear unmixing and to a lesser extent on linear regression. After the MLP models, linear regression on normalised data (bands 2357) appears to yield the best result, but it is almost equal and not significantly different from the prediction error made by the normalised linear unmixing with VIS endmembers. The simple regression model that uses the normalised near infrared band as single independent variable, however, performs worse than linear unmixing with normalised data and linear regression with four spectral bands.

Reducing the number of endmembers from three to two (vegetation versus no vegetation) has almost no impact on the outcome of linear unmixing. For normalised data, the error is not even significantly different. For MLP, the effect of reducing the number of output nodes is ambiguous because of the already small differences in error, and because reducing the number of end members sometimes decreases and sometimes increases the error, depending on the scenarios that are compared. As far as the type of endmembers for linear unmixing is concerned (VIS versus SVD), the VIS model produces the best results if the data are spectrally normalised.

## 5. Deriving urban green indicators at neighbourhood level

Cities form complex spaces with many social interactions and developments. Existing administrative or census zones do not necessary reflect this complexity, and are therefore often not suited to represent urban communities and analyse change processes. The output of the per-pixel vegetation estimates for Brussels was therefore spatially aggregated to the level of neighbourhoods. These neighbourhoods were defined by De Corte and Sanderson [63] based on 10 criteria, an existing typology of the residential environment [64] and interviews with community representatives. The Brussels capital region is covered by 118 such neighbourhoods. Additionally 6 industrial zones, 18 green and water areas, and 3 cemeteries were delineated, yielding a total of 145 urban zones. For each of these zones, total vegetation cover was calculated from the predictions made by the following models: linear regression with normalised ETM+ bands 2,3,5 and 7, linear unmixing with SVD and normalised VIS endmembers, and normalised MLP with a single output node. Zonal vegetation cover was calculated by averaging the predicted vegetation abundances of each pixel inside every neighbourhood's polygon. To compare results, the high resolution land-cover classification was used as a reference to evaluate the accuracy of the prediction models at neighbourhood level (table 3). The analysis was limited to the neighbourhoods that were fully overlapped by the land-cover map, i.e. 95 out of 145.

The results of all prediction models are highly correlated to the vegetation cover determined by the land-cover map. The lowest mean absolute error, however, was achieved by the linear regression analysis (2%), followed by the MLP (3.4%). For LSMA, the mean absolute error is much higher for both the VIS (5.5%) and SVD (4.7%) based unmixing. Except for linear regression, the mean error indicates that each model is biased towards underestimating the actual vegetation cover. Especially the LSMA model with VIS endmembers suffers from a severe negative error bias.

**Figure 4.**
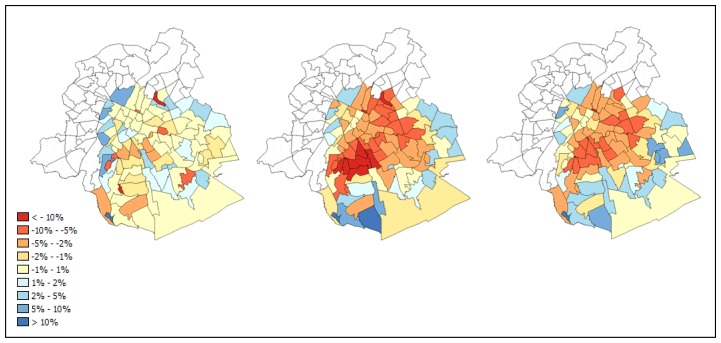
Prediction errors for linear regression (left), SVD linear unmixing (middle) and MLP model predictions (right).

Another way to compare the performance of the sub-pixel models is to count for how many neighbourhoods a particular model is the best predictor compared to the high resolution classification. The regression model performs best in 63 out of 95 cases when compared to VIS unmixing and the MLP, and in 58 cases compared to SVD unmixing and the MLP. For LSMA, particularly the VIS model performs badly with an occurrence of only 8 as the best predictor. In that respect, the SVD model scores much better. However, as its standard deviation indicates, the estimation errors are higher in cases for which it is not the best predictor. This appears to be confirmed by the maps showing prediction errors (figure 4), where high under and overestimations are apparent for certain neighbourhoods.

The spatial pattern of the estimation errors also confirms that the linear regression model is the best vegetation predictor because large errors occur for only a few neighbourhoods. Most error magnitudes are less than 0.05 and under and overestimations are both present. Underestimations occur in some urban parks (Wolvendael, Josaphat, Kruidtuin, Jubelpark, Dudenpark) while overestimations are present near the port area and the western station, a hub in the city's public transport network. The MLP and SVD unmixing also tend to estimate vegetation cover in neighbourhoods with dense forest cover rather correctly, but both make more severe underestimations in the built-up19th century belt that lies in between the central business district and the green residential areas in the southeast of the city. In the latter areas, vegetation cover tends to be overestimated. These under and overestimations are more outspoken in the linear unmixing results than in the MLP estimations.

**Figure 5.**
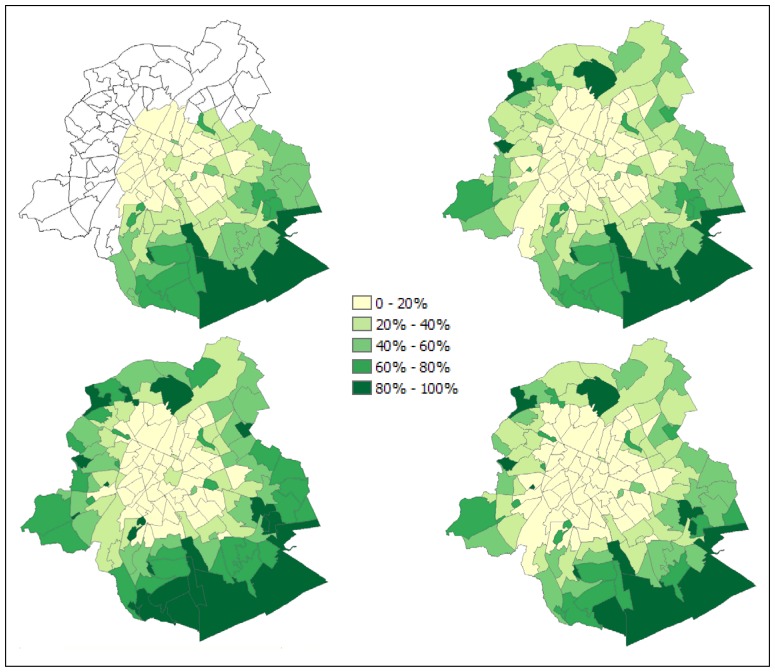
Estimations of vegetation fraction for neighbourhoods in Brussels based on a high resolution image classification with post-classification processing (top left – covers only part of the city's area), linear regression (top right) of a Landsat ETM+ image, SVD linear spectral mixture analysis (bottom left) and multi-layer perceptron unmixing (bottom right).

The derived vegetation maps (figure 5) all present a correct view of the city's structure: a sparsely vegetated, densely built-up area at the centre, and an increasing amount of green towards the city limits. The presence of some well-known landmarks is easily recognisable: the relatively green area just east of the city centre including the Warande park, the city parks in the 19^th^ century extensions surrounding the centre, the Sonian forest in the south-east, and the industrial areas along the northeast-southwest oriented canal zone.

Despite the small differences in performance between the three techniques for estimations on a per-pixel basis (table 2), the aggregation to neighbourhood level demonstrates some disparities between the patterns obtained with the three methods. When compared to the map derived from high-resolution data, the linear regression approach appears to succeed best in approximating the actual pattern of vegetation fractions. This confirms the previous results based on analysis of the errors of prediction and indicates that the most straightforward approach, linear regression, is clearly sufficient to estimate vegetation abundance at the level of neighbourhoods. For spatially more detailed estimates, MLP seems to be the most appropriate method according to the results of this study.

The predictions of vegetation abundance made by the different sub-pixel models can be used to estimate the total vegetation cover within Brussels (table 4). Administrative estimations drawn from an inventory made in 1999 arrive at a total green cover of about 8563 hectares [26]. The ETM+ image used in this study dates from the same year. Theoretically, this means that estimates made by the sub-pixel models might be compared to the administrative estimation. However, because the inventory includes green areas in a broad sense while the unmixing models estimate vegetation fraction in a strict sense, the latter will arrive at lower estimates of total vegetation cover. Indeed, while a park or garden, for instance, is considered as a green area by the inventory this does not necessarily imply a full vegetation cover as parks and gardens may contain pathways, fountains, bare soil and other non vegetative elements. Taking this into account, the sub-pixel models seem to produce realistic estimates of vegetation cover. The only exception is the VIS unmixing, which appears to underestimate vegetation. This was already indicated by the rather high mean prediction error of -5.3%. Because the mean error of the linear regression model is almost zero, which indicates that underestimations in some zones are compensated for by overestimations in other zones, this unmixing model may be considered as the most reliable estimator for total vegetation cover at city level.

## 5. Conclusions

Our results indicate that although the differences of predicting urban vegetation cover at pixel level are small among the examined mixture models, they are nevertheless statistically dissimilar in most cases. Only the various MLP models (normalised, not normalised, one, two or three output nodes) are statistically similar or almost similar, indicating that each model manages to adapt rather well to the data. Some linear models (regression and unmixing) also yielded statistically similar results among each other. The positive effect of brightness normalisation is most outspoken in linear unmixing, which suggests that it effectively reduces the spectral variability of the VIS endmembers. The normalisation appears to have no impact on the performance of the MLP models. Models with less than three endmembers also generated results that are rather similar to those produced by three endmember models.

When the vegetation predictions are spatially aggregated to the level of neighbourhoods, differences between the outcome of the models are more clearly present. Linear regression analysis on the spectrally normalised green, red and middle infrared bands appears to yield the best result, followed by the MLP model. Especially the VIS linear unmixing performs rather poorly. It has high prediction errors, and tends to underestimate vegetation cover. For the SVD model, overestimations partly compensate underestimations as the absolute prediction error is nearly the same as for VIS, but the error bias lies closer to zero.

Given these results, it appears that a simple regression model may suffice, and even produce superior predictions, to map urban vegetation at spatially aggregated levels such as neighbourhoods. At a more detailed spatial level, such as for individual pixels, the MLP approach yields the best results.

## Figures and Tables

**Table 1.** Overview of mean absolute errors and mean errors for each applied sub-pixel model, including Wilcoxon signed-rank p-values as an indication of statistically significant differences in errors. Numbers highlighted in red indicate models for which the null hypothesis is accepted with alpha = 0.01, indicating statistically identical results.

	MAE	ME	Lin. unmixing (VIS) not normalised	Lin. unmixing (VIS) normalised	Lin. unmixing (2 EM) not normalised	Lin. unmixing (2 EM) normalised	Lin. unmixing (SVD)	Lin. unmixing (modified SVD)	Lin. regres. (all bands) not normalised	Lin. regres. (bands 3,4) not normalised	Lin. regres. (bands 2357) normalised	Lin. regres. (band 4) normalised	MLP (VIS) not normalised	MLP (VIS) normalised	MLP (2 EM) not normalised	MLP (2 EM) normalised	MLP (1 EM) not normalised	MLP (1 EM) normalised
MAE			**0.1322**	**0.1090**	**0.1318**	**0.1070**	**0.3457**	**0.1270**	**0.1196**	**0.1269**	**0.1049**	**0.1124**	**0.1008**	**0.0963**	**0.0966**	**0.1000**	**0.0972**	**0.0949**
ME			**-0.0747**	**-0.0457**	**-0.0266**	**-0.0130**	**-0.3246**	**-0.0001**	**-0.0047**	**-0.0055**	**-0.0045**	**-0.0076**	**0.0079**	**0.0100**	**0.0127**	**0.0122**	**0.0117**	**0.0072**
Lin. unmixing (VIS) not normalised	**0.1322**	**-0.0747**	1	0	0.0001	0	0	0.0031	0	0.518	0	0	0	0	0	0	0	0
Lin. unmixing (VIS) normalised	**0.1090**	**-0.0457**	0	1	0	0.2378	0	0	0	0	0.0738	0	0.0799	0	0	0.001	0	0
Lin. unmixing (2 EM) not normalised	**0.1318**	**-0.0266**	0.0001	0	1	0	0	0.0349	0	0.0108	0	0	0	0	0	0	0	0
Lin. unmixing (2 EM) normalised	**0.1070**	**-0.0130**	0	0.2378	0	1	0	0	0	0	0.0078	0	0.0097	0	0	0.0002	0	0
Lin. unmixing (SVD)	**0.3457**	**-0.3246**	0	0	0	0	1	0	0	0	0	0	0	0	0	0	0	0
Lin. unmixing (modified SVD)	**0.1270**	**-0.0001**	0.0031	0	0.0349	0	0	1	0.3035	0	0	0.0017	0	0	0	0	0	0
Lin. regres. (all bands) not normalised	**0.1196**	**-0.0047**	0	0	0	0	0	0.3035	1	0	0	0	0	0	0	0	0	0
Lin. regres, (bands 3,4) not normalised	**0.1269**	**-0.0055**	0.518	0	0.0108	0	0	0	0	1	0	0	0	0	0	0	0	0
Lin. regres. (bands 2357) normalised	**0.1049**	**-0.0045**	0	0.0738	0	0.0078	0	0	0	0	1	0	0.0005	0	0	0	0	0
Lin. regres. (band 4) normalised	**0.1124**	**-0.0076**	0	0	0	0	0	0.0017	0	0	0	1	0	0	0	0	0	0
MLP (VIS) not normalised	**0.1008**	**0.0079**	0	0.0799	0	0.0097	0	0	0	0	0.0005	0	1	0	0	0.0037	0	0
MLP (VIS) normalised	**0.0963**	**0.0100**	0	0	0	0	0	0	0	0	0	0	0	1	0.0208	0	0.9259	0.0053
MLP (2 EM) not normalised	**0.0966**	**0.0127**	0	0	0	0	0	0	0	0	0	0	0	0.0208	1	0	0	0.0039
MLP (2 EM) normalised	**0.1000**	**0.0122**	0	0.001	0	0.0002	0	0	0	0	0	0	0.0037	0	0	1	0	0
MLP (1 EM) not normalised	**0.0972**	**0.0117**	0	0	0	0	0	0	0	0	0	0	0	0.9259	0	0	1	0.1892
MLP (1 EM) normalised	**0.0949**	**0.0072**	0	0	0	0	0	0	0	0	0	0	0	0.0053	0.0039	0	0.1892	1

**Table 2.** Linear regression models ordered by decreasing amount of explained variance.

No brightness normalisation			Brightness normalisation		
ETM+ bands	R^2^_adj_	Mean absolute error	ETM+ bands	R^2^_adj_	Mean absolute error
1,2,3,4,5,7	0.811	0.120	2,3,5,7	0.851	0.105
1,3,4,5,7	0.811	0.120	2,3,4,5,7	0.851	0.105
3,4,5,7	0.808	0.121	2,3,4,7	0.850	0.105
3,4,7	0.800	0.123	3,4,7	0.844	0.107
3,4	0.791	0.127	3,4	0.836	0.110
4	0.536	0.208	4	0.828	0.112

**Table 3.** Comparison of urban green estimates made by the three medium resolution sub-pixel prediction models to the high resolution land-cover map. Analysis is limited to image overlap (95 out of 145 neighbourhoods).

	Linear regression	Linear unmixing		MLP
		VIS	SVD	
**# neighbourhoods**	95	95	95	95
**Correlation**	0.992	0.989	0.978	0.990
**Mean absolute error**	0.020	0.055	0.047	0.034
**Mean error**	0.000	-0.053	-0.029	-0.014
**Standard deviation**	0.031	0.035	0.041	0.041
**95% conf. interval**	± 0.006	± 0.007	± 0.008	± 0.008
**# times best predictor with VIS**	63 (66.3%)	8 (8.4%)		24 (25.3%)
**# times best predictor with SVD**	58 (61.1%)		18 (18.9%)	19 (20.0%)

**Table 4.** Total amount of vegetation cover in Brussels as predicted by the four sub-pixel estimation models.

	Linear regression	Linear unmixing		MLP
		VIS	SVD	
**# neighbourhoods**	145	145	145	145
**Total vegetated area (ha)**	7114.63	6198.92	6848.69	7004.95
